# The Warburg Effect in Yeast: Repression of Mitochondrial Metabolism Is Not a Prerequisite to Promote Cell Proliferation

**DOI:** 10.3389/fonc.2020.01333

**Published:** 2020-08-19

**Authors:** Cyrielle L. Bouchez, Noureddine Hammad, Sylvain Cuvellier, Stéphane Ransac, Michel Rigoulet, Anne Devin

**Affiliations:** ^1^CNRS, Institut de Biochimie et Génétique Cellulaires, UMR 5095, Bordeaux, France; ^2^Univ. de Bordeaux, Institut de Biochimie et Génétique Cellulaires, UMR 5095, Bordeaux, France

**Keywords:** Warburg effect, mitochondria, oxidative phosphorylation, mitochondrial biogenesis, Hap4p, yeast

## Abstract

O. Warburg conducted one of the first studies on tumor energy metabolism. His early discoveries pointed out that cancer cells display a decreased respiration and an increased glycolysis proportional to the increase in their growth rate, suggesting that they mainly depend on fermentative metabolism for ATP generation. Warburg's results and hypothesis generated controversies that are persistent to this day. It is thus of great importance to understand the mechanisms by which cancer cells can reversibly regulate the two pathways of their energy metabolism as well as the functioning of this metabolism in cell proliferation. Here, we made use of yeast as a model to study the Warburg effect and its eventual function in allowing an increased ATP synthesis to support cell proliferation. The role of oxidative phosphorylation repression in this effect was investigated. We show that yeast is a good model to study the Warburg effect, where all parameters and their modulation in the presence of glucose can be reconstituted. Moreover, we show that in this model, mitochondria are not dysfunctional, but that there are fewer mitochondria respiratory chain units per cell. Identification of the molecular mechanisms involved in this process allowed us to dissociate the parameters involved in the Warburg effect and show that oxidative phosphorylation repression is not mandatory to promote cell growth. Last but not least, we were able to show that neither cellular ATP synthesis flux nor glucose consumption flux controls cellular growth rate.

## Introduction

Cell proliferation requires anabolic pathways that require energy for their accomplishment. ATP is the central molecule in energy conversion processes that can be synthesized through two kinds of reaction: substrate-level phosphorylation (glycolysis and Krebs cycle) and mitochondrial oxidative phosphorylation. Mitochondria has long been proposed to play a central role in ATP turn-over since they synthesize most of the cellular ATP. It is well-known that the energetic yield of both pathways is very different since when one glucose is being oxidized, glycolysis will generate 2 ATP, whereas mitochondria can generate over ten times more but requires oxygen, the final electron acceptor of the respiratory chain.

O. Warburg conducted one of the first studies on tumor energy metabolism. His early discoveries showed that cancer cells display a decreased respiration and an increased glycolysis proportional to the increase in their growth rate, suggesting that they mainly depend on fermentative metabolism for ATP generation (1). Because the repression of oxidative metabolism occurs even if oxygen is plentiful, this metabolic phenomenon was named “aerobic glycolysis.” Warburg later proposed that dysfunctional mitochondria are the root of aerobic glycolysis (2) and further hypothesized that this event is the primary cause of cancer. It should be stressed here that the Warburg effect, as defined by O. Warburg, requires the concomitant and correlated variations of the three parameters mentioned above: respiratory rate, glycolysis and growth rate. If one of these parameters does not evolve as expected, one can no longer consider the observed phenomenon to be a Warburg effect. Warburg's results and hypothesis generated controversies that are persistent to this day. Moreover, this effect is very different from the Crabtree effect, which is defined as the inhibition of cellular oxygen consumption upon glucose addition to cells and fall under a kinetic regulation of mitochondrial oxidative phosphorylation (3).

It has been shown that oxidative phosphorylation activity can be increased in cultured cancer cells (4), which implies that the decrease in mitochondrial activity in these cells is not irreversible. In the same line of thought, it has been shown that some cancer cells, when cultured in petri dishes, can reversibly switch between fermentation and oxidative metabolism, depending on the absence or the presence of glucose and the environmental conditions (4–6). Another hypothesis was that the down regulation of oxidative phosphorylation was used by cancer cells to proliferate in hypoxic environments. Nonetheless, a considerable body of evidence challenges the paradigm of the purely “glycolytic” cancer cell (7). Some glioma, hepatoma and breast cancer cell lines possess functional mitochondria and they obtain their ATP mainly from oxidative phosphorylation (8–11). Moreover, a model proposed that “glycolytic” cells could establish a metabolic symbiosis with the “oxidative” ones through lactate shuttling (12). This points out that the metabolic plasticity observed *in vitro* may have an impact on tumor physiology *in vivo*.

Therefore, it is crucial to understand the mechanisms by which cancer cells can reversibly regulate the two pathways of their energy metabolism as well as the functioning of this metabolism in cell proliferation. Both pathways are thermodynamically controlled by two forces: the phosphate potential (ΔGp) and the redox potential (ΔG_redox_), which implies that they are inherently linked. Further, they are kinetically regulated by metabolites that can arise from one another (13, 14). These pathways being so intertwined raises the question whether the downregulation of mitochondrial metabolism is a mandatory step to increase the flux through glycolysis.

A more finalist way of analyzing the Warburg effect is to investigate its added value to promote cell growth. A review of the literature shows that the possible benefits of this energy metabolism rewiring for cancer cell growth are not clear. Indeed, it is well-known that aerobic glycolysis is inefficient in terms of ATP synthesis yield when compared with mitochondrial respiration (15–17). However, in mammals, the rate of glucose metabolism through aerobic glycolysis is 10–100 higher than complete oxidation of glucose by mitochondrial oxidative phosphorylation. One could thus hypothesize that one of the functions of the Warburg effect would be to allow the rapid production of ATP that can be rapidly tuned to answer the demand for ATP synthesis. It has also been proposed that intense aerobic glycolysis was necessary to ensure the supply of glycolytic intermediates as building blocks to biosynthetic pathways. To this day, and despite numerous studies, the possible advantages of this metabolic rewiring are still under investigation.

There is a number of limitations to the use of cancer cells in culture in order to study their energy metabolism. First and foremost, in these cells, the metabolic deviation is already in place, not allowing a study of the molecular mechanisms giving rise to that deviation. Second, most of the studies conducted in these cells are conducted under hyperglycemic (22.5 mM glucose) and hyperoxic (21% O_2_) conditions. These are two crucial parameters when one considers cell energy metabolism, glucose feeding glycolysis and oxygen being the electrons acceptor in the respiratory chain. From a metabolic point of view, the fermenting yeast Saccharomyces cerevisiae and tumor cells share several features (18, 19). In both cell types, there are mechanisms that enhance glycolytic flux concomitantly with the repression of oxidative phosphorylation in the presence of glucose, and fermentation is preferred even in the presence of oxygen. Here, we made use of yeast as a model to study the Warburg effect and its eventual function in supporting an increased ATP demand to support cell proliferation. The role of oxidative phosphorylation repression in this effect was investigated. We show that yeast is a good model to study the Warburg effect, where all parameters can be reconstituted. Moreover, contrary to what was proposed by Warburg and in accordance with a number of reports from the literature, we show that in this model mitochondria are not dysfunctional: there are fewer mitochondria respiratory chain units per cell. Identification of the molecular mechanisms involved in this process allowed us to dissociate the parameters involved in the Warburg effect and show that oxidative phosphorylation repression is not mandatory to favor cell growth. Last but not least, we were able to show that cellular ATP synthesis flux does not control cellular growth rate.

## Experimental Procedures

### Yeast Strains, Plasmids, and Culture Medium

The following yeast strains were used in this study: BY4742 (MATα; his3Δ1; leu2Δ0; lys2Δ0; ura3Δ0); BY4742 Δ*hap4* (MATα; his3Δ1; leu2Δ0; lys2Δ0; ura3Δ0; hap4:: kanMX4); BY4742 Δ*hxk2* (MATα; his3Δ1; leu2Δ0; lys2Δ0; ura3Δ0; Hxk2:: kan MX4); *Candida utilis* (CBS621) and *Saccharomyces cerevisiae* (yeast foam). Strains Δhxk2-Hap4p↑ and Δhap4-Hap4p↑ are BY4742 strains previously described (14).

Cells were grown aerobically at 28°C in the following medium: 0.175% yeast nitrogen base (Difco), 0.2% casein hydrolysate (Merck), 0.5% (NH_4_)_2_SO_4_, 0.1% KH_2_PO_4_, 2% lactate (w/v) (Prolabo), pH 5.5, 20 mg.L^−1^ L-tryptophan (Sigma), 40 mg.L^−1^ adenine hydrochloride (Sigma) and 20 mg.L^−1^ uracil (Sigma). When cells carried a plasmid, uracil was omitted in the growth medium [pTET-HAP4 (20, 21)]. Where indicated, glucose (60 mM) was added to the medium. Growth was measured at 600 nm in a Safas spectrophotometer (Monaco) in a 1 mL cuvette. Dry weight determinations were performed on samples of cells harvested throughout the growth period, washed twice in distilled water and weighed after extensive drying.

### Oxygen Consumption Assays

Oxygen consumption was measured polarographically at 28°C using a Clark oxygen electrode in a 1 mL thermostatically controlled chamber. 1 mL of culture was transferred to the chamber and respiratory rates (JO_2_) were determined from the slope of a plot of O_2_ concentration vs. time. The measured activities are normalized per mg dry weight. Respiration assays of growing cells were performed in the growth medium. In the case of uncoupled respiratory rate (10 μM CCCP), 100 mM Ethanol was added to the culture medium (22).

### Glucose Measurement

Cells were grown in 2% lactate synthetic complete medium. Each hour, 1 mL of the culture was harvested and centrifuged. Culture supernatant was heated at 80°C for 5 min. Glucose was quantified in the supernatant with the Megazyme “D-Glucose HK assay Kit.”

### Ethanol Measurement

Cells were grown in 2% lactate synthetic complete medium. Each hour, 1 mL of the culture was harvested and centrifuged. Culture supernatant was mixed with 25% of PCA. Then, KOMO (KOH 2M, MOPS 0.3M) was added to adjust the pH to 7. Samples were diluted in potassium phosphate buffer (50 mM) pH9 containing NAD^+^ (2 mM), Aldehyde dehydrogenase (0.3 U/mL) and alcohol dehydrogenase (70 U/mL). Ethanol was quantified by NAD^+^ reduction at 340 nm and 28°C.

### Cytochrome Content Determination

The cellular content of mitochondrial cytochromes c+c_1_, b and a+a_3_ was calculated as described in Dejean et al. (23) considering the respective molar extinction coefficient values and the reduced-minus-oxidized spectra recorded using a dual beam spectrophotometer (Varian, cary 4000).

### Enzymatic Activities Determination

Cells were washed and then broken by vigorous shaking with an equal volume of glass beads in a buffer containing 50 mM Tris-HCl pH 7.4 and a mixture of protease inhibitors (Complete EDTA-freeTM, Roche). Centrifugations (700 g, 2 min) allowed the elimination of pelleted unbroken cells and glass beads. Cellular proteins were quantified by the Lowry method. Citrate synthase (2.3.3.1) activity was determined by monitoring at 412 nm the oxidation of coenzyme A (produced by citrate synthase activity) by 5,5'-dithiobis-2-nitrobenzoic acid (DTNB) as a function of time, in a Safas spectrophotometer. The enzyme activity was calculated using an extinction coefficient of 13 600 M^−1^.cm^−1^ at 412 nm. One citrate synthase unit was equal to 1 μmole of DTNB reduced per minute per mg dry weight. Cytochrome c oxidase activity (1 mM potassium cyanide–sensitive) was determined by monitoring spectrophotometrically (550 nm) the rate of disappearance of reduced cytochrome c in the following buffer: 50 mM PiK, 100 μM reduced cytochrome c. The enzyme activity was determined using an extinction coefficient of 18,500 M^−1^.cm^−1^ at 550 nm for reduced cytochrome c (all from Sigma). Hexokinase activity was determined by monitoring the rate of NAD^+^ reduction spectrophotometrically (340 nm) (2 mM) in the presence of 0.5 U/mL glucose-6-phosphate dehydrogenase (G6PDH), 10 mM glucose, 1 mM ATP, 1 mM MgCl_2_.

### Protein Extraction, Electrophoresis, and Western-Blot

Cells were lysed using a mixture of 7.5% β-mercaptoethanol in 1.85M NaOH. After 10 min incubation on ice, proteins were precipitated by the addition of an equal volume of 3M trichloroacetic acid for 10 min on ice. After a rapid centrifugation at 4°C, the protein pellet was suspended in a mixture of 10% SDS and sample buffer (0.06M Tris, 2% SDS, 2% ß-mercaptoethanol, 5% glycerol, 0.02% bromophenol blue). Protein amounts corresponding to 0.5 OD units of cells were separated by 10% SDS-PAGE performed according to the method of Laemmli (24). After electro-transfer onto nitrocellulose membranes (Amersham Biosciences), proteins were probed with the desired primary antibodies: α-Hap4p (see below) and α-PGK1 (monoclonal antibody, Invitrogen) and detected using peroxidase-conjugated secondary antibodies (Jackson ImmunoResearch) ECL Prime reagent (Amersham Biosciences), according to the manufacturer instructions. Signal quantifications were done using the ImageJ software.

### Antibodies

Polyclonal anti-Hap4p antibodies were generated by Eurogentec using the Hap4p fragment 330–554 as an antigen. Phospho-glycerate kinase antibody was a commercial antibody (PGK1; Invitrogen).

### Statistical Analysis

Results are expressed as mean ± SD. Statistical analysis was carried out using ANOVA test for all results. Prism software (GraphPad, San Diego, CA) was used for all tests. A *p* < 0.05 was considered significant. A *p* < 0.05 was considered significant and ^*^p < 0.05; ^**^p < 0.01; ^***^p < 0.001; ^****^p < 0.0001.

## Results and Discussion

### Yeast as a Model to Study the Warburg Effect

**Figure 1:**
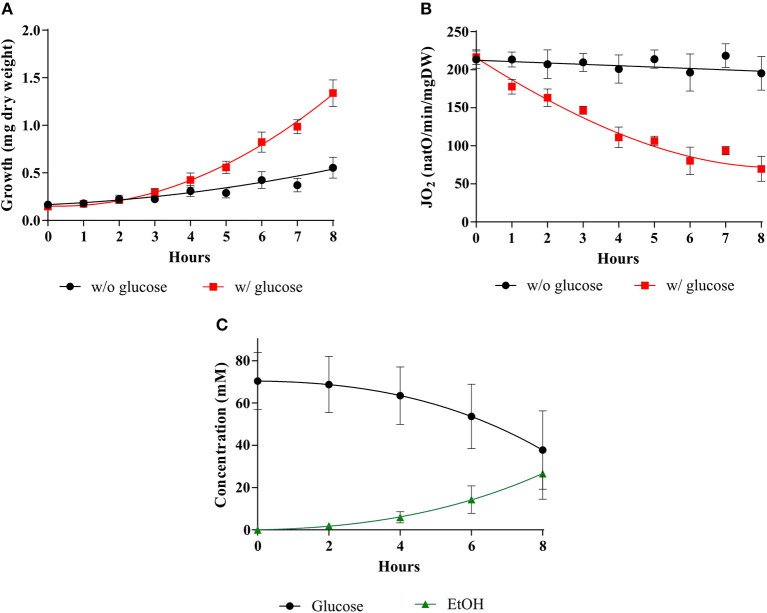
Induction of the Warburg effect in *S. cerevisiae*. The growth medium of *S. cerevisiae* was supplemented with (w/) 60 mM of glucose at T0 (■) or without (w/o) (•). **(A)** For each condition, growth was followed for 8 h. Results shown are means of at least 10 separate experiments ± SD. **(B)** For each condition, the respiratory rate was followed for 8 h. Results shown represent means of at least 10 separate experiments ± SD. **(C)** Glucose consumption (•) and ethanol (EtOH) production (▲) were quantified. Results shown represent means of at least seven separate experiments ± SD.

As stated above, in his seminal paper, Warburg showed a tight link between cell proliferation, cell respiration repression and aerobic glycolysis stimulation. Thus, what is now referred to as the Warburg effect must rely on these three parameters and their relationship(s). In order to define whether yeast would be a good model to study the induction of the Warburg effect, these three parameters were assessed in the presence or in the absence of glucose during yeast growth on non-fermentable medium. Figure 1 shows that whereas in the absence of glucose both growth and respiration are constant, upon glucose addition, cellular growth is significantly increased (A). This is associated with a decrease in cellular respiratory rate (B) and an increase in glucose fermentation (C). Altogether, these parameters evolution upon glucose addition shows that yeast is a suitable model for a kinetic study of the Warburg effect induction.

**Figure 2:**
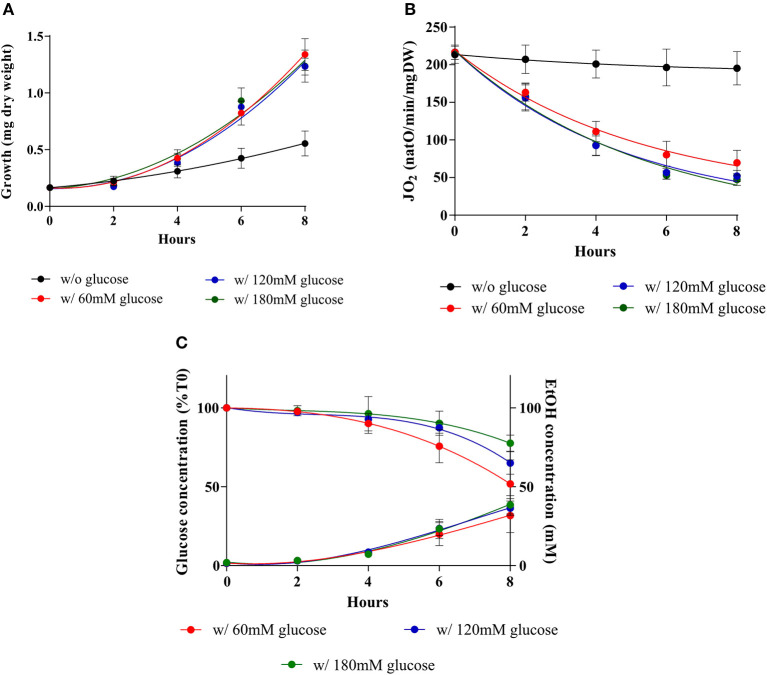
Induction of the Warburg effect in *S. cerevisiae* at various external glucose concentrations. *S. cerevisiae* growth medium was supplemented with 60 mM (•), 120 mM (•), 180 mM (•) of glucose or not (•) at T0. **(A)** For each condition, growth was followed for 8 h. Results shown represent means of at least seven separate experiments ± SD. **(B)** The respiratory rate was followed for 8 h. Results shown represent means of at least seven separate experiments ± SD. **(C)** After addition of the different glucose concentrations in the medium culture, glucose consumption and ethanol production were quantified. Results shown represent means of at least four separate experiments ± SD.

As shown in Figure 1, during the 8-h kinetic measurement up to 30 mM in glucose are consumed. 60 mM glucose was thus the lowest concentration used to ensure that glucose would not run low under our experimental conditions. To assess an eventual role of glucose concentration on the induction of the Warburg effect cellular growths, respiratory rates and glucose consumptions were assessed upon addition of three distinct glucose concentrations. Figure 2 shows that upon glucose addition, cellular growth is increased in a comparable extent whichever the glucose concentration (A). This is associated with a decrease in cellular respiratory rate to a comparable extent whichever the glucose concentration (B) and glucose fermentation to a comparable extent whichever the glucose concentration, after 8 h about 30 mM of glucose were consumed whichever the initial glucose concentration (C).

### Mitochondria Are Not Dysfunctional, There Are Fewer Mitochondria

**Figure 3:**
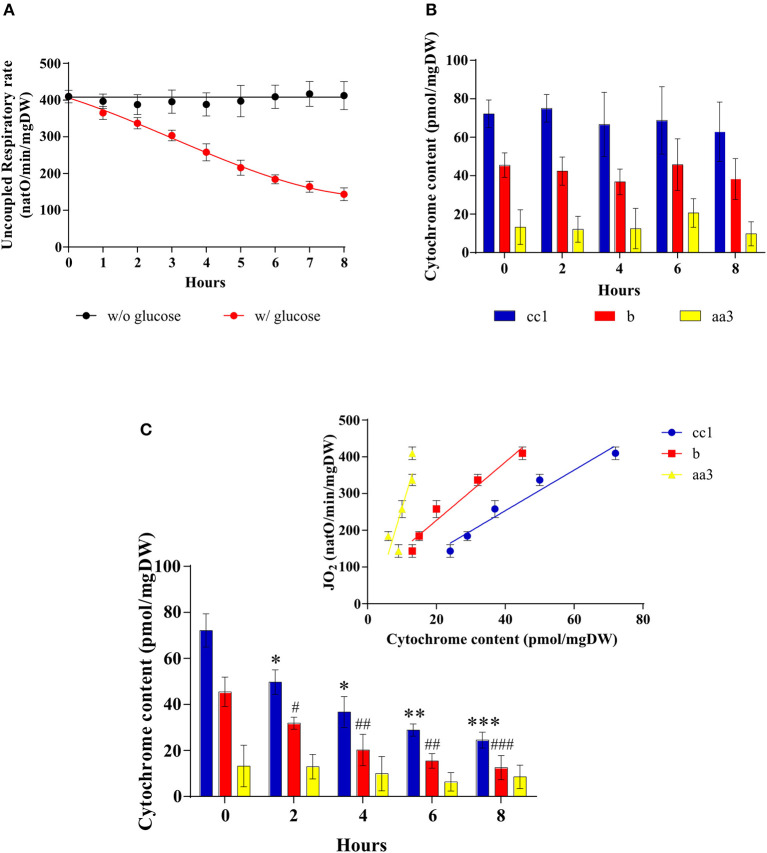
Mitochondrial amount after induction of the Warburg effect in *S. cerevisiae*. The growth medium of *S. cerevisiae* was supplemented with 60 mM of glucose at T0 (•) or not (•). **(A)** The uncoupled respiratory rate was followed for 8 h. Results shown represent means of at least five separate experiments ± SD. **(B)** Cytochrome content was quantified every 2 h in cells in absence of glucose in the medium. Results shown represent means of at least three separate experiments ± SD. **(C)** Cytochrome content was quantified every 2 h in cells after glucose addition in the medium. Inset: A linear regression between respiratory rate and cytochrome content is presented for each cytochrome. Results shown represent means of at least four separate experiments ± SD. For each condition values are compared to the corresponding T0 and the *p*-value is represented as * for cc1; # for b.

A decrease in cellular respiratory rate can originate in a number of distinct processes such as an alteration of mitochondria [i.e., dysfunctional mitochondria as proposed by Warburg (2, 25)], a modulation of the respiratory state (such as a decrease in phosphorylating processes), or a decrease in cellular mitochondrial content (herein assimilated to the content in respiratory chain units). In order to pinpoint the origin of the glucose-induced decrease in respiratory rate, a number of mitochondrial parameters were assessed. Figure 3A shows that whereas in the absence of glucose the uncoupled cellular respiratory rate is stable throughout exponential growth of the cells, glucose addition induces a decrease of this rate, i.e., a decrease in respiratory chain activity. Because this decrease in respiratory chain activity can be due to either a kinetic regulation of the respiratory chain or a quantitative decrease in respiratory chain complexes, we quantified mitochondrial cytochromes within the cells. In the absence of glucose, the cytochromes are stable throughout exponential growth of the cells (Figure 3B). When the Warburg effect was induced (glucose addition) a continuous decrease of all types of cytochromes was assessed (Figure 3C). Moreover, the decrease in respiratory rate is directly proportional to the decrease in mitochondrial cytochromes (inset), showing that this oxygen consumption decrease is actually due to a decrease in mitochondrial respiratory chain units within the cells. This decrease in mitochondrial content was further confirmed by measuring mitochondrial enzymatic activities i.e., citrate synthase and cytochrome c oxidase. Both were decreased upon glucose addition to cells (Table 1). However, it should be stressed here that citrate synthase activity modulation should be interpreted with caution in yeast where a peroxisomal citrate synthase exists and is downregulated by glucose (26–28).

**Table 1:** Citrate synthase and cytochrome c oxidase activities in wild type and mutant cells.

		**T0**	**T2**		**T4**		**T6**		**T8**	
		**–**	**–**	**+**	**–**	**+**	**–**	**+**	**–**	**+**
*S. c* – WT	CS	0.37 (± 0.2)	0.34 (± 0.19)	0.19 (± 0.1)**	0.30 (± 0.16)	0.08 (± 0.04)**	0.33 (± 0.17)	0.04 (± 0.01)**	0.33 (± 0.17)	0.04 (± 0.02)**
	COX	0.07 (± 0.02)	0.06 (± 0.02)	0.04 (± 0.01)	0.06 (± 0.02)	0.03 (± 0.01)**	0.05 (± 0.01)	0.02 (± 0.01)**	0.05 (± 0.02)	0.03 (± 0.01)*
*S. c* – Δ*hxk2*	CS	0.39 (± 0.12)	0.48 (± 0.05)	0.28 (± 0.09)**	0.4 (± 0.14)	0.15 (± 0.05)***	0.44 (± 0.1)	0.1 (± 0.03)***	0.56 (± 0.13)	0.08 (± 0.03)***
	COX	0.09 (± 0.03)	0.09 (± 0.03)	0.06 (± 0.02)	0.08 (± 0.02)	0.05 (± 0.01)*	0.09 (± 0.03)	0.05 (± 0.02)*	0.1 (± 0.03)	0.05 (± 0.02)*
*S. c* – Δ*hap4↑*	CS	0.28 (± 0.03)	0.35 (± 0.03)	0.23 (± 0.03)*	0.32 (± 0.07)	0.23 (± 0.02)	0.32 (± 0.04)	0.23 (± 0.05)*	0.35 (± 0.01)	0.22 (± 0.06)*
	COX	0.14 (± 0.04)	0.16 (± 0.04)	0.14 (± 0.03)	0.19 (± 0.04)	0.15 (± 0.02)	0.18 (± 0.02)	0.18 (± 0.05)	0.18 (± 0.02)*	0.14 (± 0.05)
*S. c* – Δ*hxk2hap4↑*	CS	0.55 (± 0.06)	0.58 (± 0.09)	0.55 (± 0.06)*	0.51 (± 0.05)	0.38 (± 0.05)*	0.53 (± 0.05)	0.35 (± 0.08)**	0.57 (± 0.02)*	0.31 (± 0.05)*
	COX	0.1 (± 0.03)	0.1 (± 0.03)	0.1 (± 0.03)	0.11 (± 0.03)	0.1 (± 0.04)	0.13 (± 0.03)	0.11 (± 0.04)	0.13 (± 0.02)	0.12 (± 0.03)
*C. u*	CS	0.09 (± 0.04)	0.1 (± 0.04)	0.08 (± 0.03)	0.11 (± 0.05)	0.08 (± 0.04)	0.1 (± 0.03)	0.09 (± 0.02)	0.13 (± 0.04)	0.1 (± 0.02)
	COX	0.11 (± 0.03)	0.09 (± 0.03)	0.08 (± 0.03)	0.11 (± 0.03)	0.08 (± 0.01)	0.1 (± 0.04)	0.1 (± 0.03)	0.12 (± 0.04)	0.09 (± 0.04)

*CS, citrate synthase; COX, cytochrome oxidase. Enzymatic activities were measured as stipulated in the Materials and Methods section and is expressed in μmol/min/mg protein. (**–**) indicates in the absence of glucose. (+) indicates in the presence of glucose. T0-2-4-6-8 are the hours of incubation w/ or w/o glucose. Results shown are means of at least three separate experiments ± SD*.

### The Decrease in Mitochondrial Amount Originates in a Decrease in Mitochondrial Biogenesis

**Figure 4:**
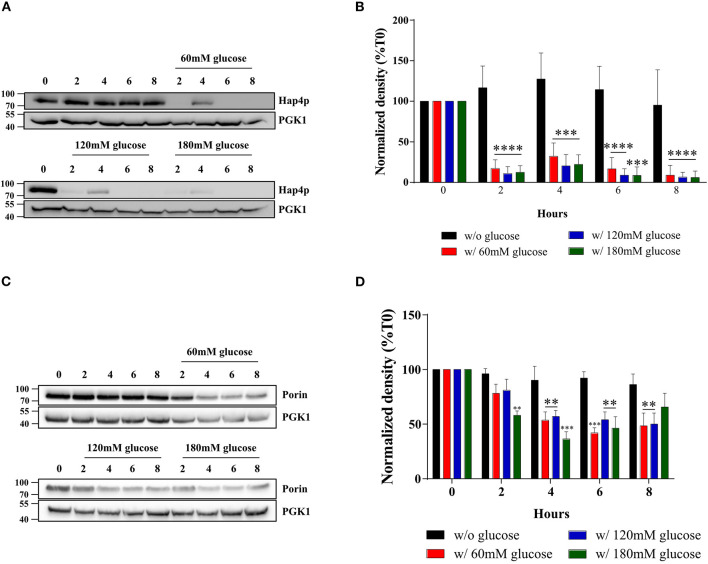
Hap4p and porin amount after induction of the Warburg effect in *S. cerevisiae*. The growth medium of *S. cerevisiae* was supplemented with 60, 120, and 180 mM of glucose or not at T0. **(A)** The relative levels of the co-activator Hap4p were assessed every 2 h. Total protein extracts were analyzed by SDS-PAGE and western blot using antibodies directed against Hap4p and phospho-glycerate kinase (PGK1) as a loading control. **(B)** Results representative of at least four experiments are shown. Hap4p signal was quantified, signal intensity was normalized to PGK1 and expressed in percentage of T0 for each experiment. The bar-graph shows mean % of T0 ± SD for all experiments. **(C)** The relative levels of a mitochondrial protein, Porin, were assessed every 2 h. Total protein extracts were analyzed by SDS-PAGE and western blot using antibodies directed against porin and phospho-glycerate kinase (PGK1) as a loading control. **(D)** Results representative of at least four experiments are shown. Porin signal was quantified, signal intensity was normalized to PGK1 signal and expressed in percentage of T0 for each experiment. The bar-graph shows mean % of T0 ± SD for all experiments.

The amount of mitochondria within a cell is controlled by its turnover *i.e.*, the respective rates of mitochondrial biogenesis and mitochondrial degradation. The HAP complex has been shown to be involved in the specific induction of genes involved in gluconeogenesis, metabolism of alternate carbon sources, respiration, and mitochondrial development. Indeed, the disruption of any subunit of this complex renders the cells unable to grow on non-fermentable carbon sources (29–32). Moreover, many genes involved in energy metabolism have been shown to be regulated by this complex (33–35). In order to determine whether the biogenesis of the mitochondrial compartment was affected upon glucose addition to the cells, we assessed the cellular amount of the master regulator of the activity of this multicomplex, the subunit Hap4p. Figure 4A shows that Hap4p amount was decreased upon glucose addition, independently of glucose concentration. This protein tends to increase after 4 h in the presence of glucose and goes back to low levels for the 6 and 8 h' time points. Quantitation of the western blots for this protein shows that is it almost undetectable after induction of the Warburg effect (Figure 4B). Further, porin, a mitochondrial outer membrane protein is shown to decrease, albeit to a lower level than the mitochondrial cytochromes, upon glucose addition to cells, independently of the glucose concentration used (Figures 4C,D). PGK1, a cytosolic enzyme was used as a loading control. This confirms the decrease in cellular mitochondrial content within the cells upon glucose addition. Further experiments showed no degradation (mitophagy) of the mitochondrial compartment upon glucose addition to cells (data not shown).

### Repression of Mitochondrial Metabolism Is Not a Prerequisite to Promote Cell Proliferation

**Figure 5:**
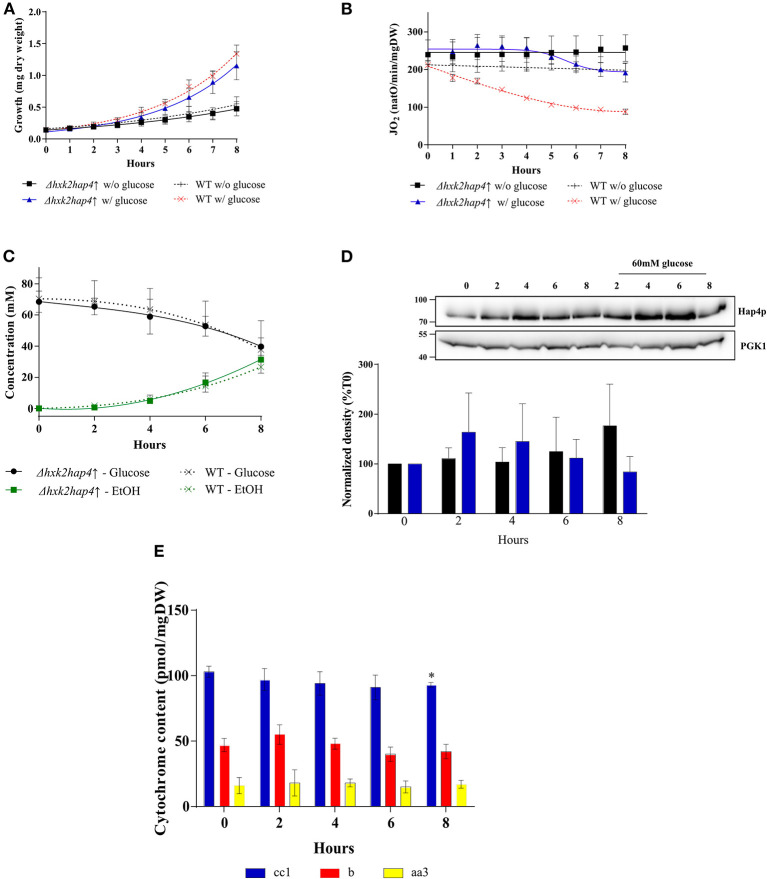
Induction of the Warburg effect in *S. cerevisiae* Δ*hxk2hap4*↑. A *S. cerevisiae* strain deleted for *hxk2* (Δ*hxk2*) was transformed with pCM189-Hap4p (Δ*hxk2hap4*↑). The growth medium was supplemented with 60 mM of glucose at T0 (▲ Δ*hxk2hap4*↑) and (x WT) or not (■Δ*hxk2hap4*↑) and (+ WT). **(A)** Growth was followed for 8 h. Results shown represent means of at least 10 separate experiments ± SD. **(B)** The respiratory rate was followed for 8 h. Results shown represent means of at least 10 separate experiments ± SD. **(C)** Glucose consumption (• Δ*hxk2hap4*↑) and (x WT) and ethanol production (• Δ*hxk2hap4*↑) and (x WT) were quantified. Results shown represent means of at least five separate experiments ± SD. **(D)** The relative levels of the co-activator Hap4p were determined every 2 h. Total protein extracts were analyzed by SDS-PAGE and western blot using antibodies directed against Hap4p and phospho-glycerate kinase (PGK1) as a loading control. Inset: Results representative of at least four experiments are shown. Hap4p signal was quantified, signal intensity was normalized to PGK1 signal and expressed as percentage of T0 for each experiment. The bar-graph shows mean % of T0 ± SD for all experiments. **(E)** Cytochrome content was quantified every 2 h. Results shown represent means of at least four separate experiments ± SD.

To further investigate the role of the decrease in mitochondrial respiratory rate on the Warburg effect we explored means to prevent mitochondrial biogenesis decrease upon glucose addition. First, we envisioned limiting the glycolytic flux. Hexokinase is the first enzyme of the glycolysis pathway. Among the three isoenzymes in both yeast and mammalian cells, hexokinase2 (Hxk2p) is the predominant hexokinase during growth on glucose. It is the yeast homologous of glucokinase (overexpressed in tumor cells) and has a dual function being both a catalyst in the cytosol and an important regulator of the glucose repression signal in the nucleus (36–39). The consequences of deleting this enzyme on the induction of the Warburg effect was thus investigated. No significant effect was observed on growth or glucose consumption (Supplementary Figures 1A,C), indicating that Hxk2p isoenzymes' carry out the glucose phosphorylating function. This was further confirmed by measuring Hxk activity in our cells (Table 2). Of note, Hxk activity was much more increased after glucose addition in Δhxk2 and Δ*hap4*↑ cells, indicating a stronger activation of Hxk2p isoenzymes by glucose in the absence of Hxk2p. The deletion of Hxk2p associated with Hap4p overexpression (see below) restores this activity to a wild-type level suggesting a repression of these enzymes by Hap4p. Deletion of Hxk2p allowed a decrease in mitochondrial oxygen consumption and in mitochondrial enzymatic activities (Table 1) upon glucose addition, this decrease was smaller than in the wild type cells (Supplementary Figure 1B). However, this deleted strain was not sufficient to prevent the induction of the Warburg effect. Since Hap4p was shown to be strongly decreased upon glucose addition to cells (see Figure 4A), we ectopically overexpressed this protein, hoping to maintain mitochondrial biogenesis in the presence of glucose. No significant effect was observed on growth or glucose consumption/fermentation (Supplementary Figures 2A,C). However, although a decrease in mitochondrial oxygen consumption and in mitochondrial enzymatic activities (Table 1) upon glucose addition was observed with the overexpression of Hap4p (Δ*hap4*↑), this decrease was smaller than in the wild type cells, with a delay observed in its induction (Supplementary Figure 2B). Next, this protein was overexpressed in the Δ*hxk2* strain (Δ*hxk2hap4p*↑, Figure 5). Considering the function of both Hxk2p and Hap4p, such a strain should exhibit an increased mitochondrial content and a strong orientation of energetic metabolism toward respiratory metabolism, altering the relationship between glycolysis and respiratory rate. In the absence of glucose, this strain exhibits a slight increase in growth (Figure 5A, data not shown) and an increase in cellular respiratory rate of about 25% (Figure 5B). Glucose addition to this strain led to an increase in proliferation comparable to the one in the wild type strain (Figure 5A). However, regarding the respiratory rate of this strain in the presence of glucose, only a slight decrease was assessed after 4 h delay and, more importantly, after 8 h, its respiratory rate was comparable to the one in the wild type strain in the absence of glucose (Figure 5B), indicating a strong maintenance of mitochondrial function even in the presence of glucose. Despite this high respiratory rate, the Δ*hxk2hap4p*↑ strain exhibits a significant -and comparable to the wild type- glucose consumption and ethanol production upon glucose addition, in accordance with the hxk activity exhibited in this strain (Figure 5C & Table 2). The ectopically overexpressed Hap4p was shown to be stable over time both in the presence and absence of glucose (Figure 5D). The origin of the maintenance of the respiratory chain activity in the presence of glucose was investigated by means of quantifying the mitochondrial cytochromes within the cells. Figure 5E shows that in this strain and in the presence of glucose, mitochondrial cytochromes are stable throughout the cell's growth. Of note, the cytochrome content in this strain in the absence of glucose is higher than in the wild type strain, in agreement with the increase in cellular respiration in absence of glucose. Mitochondrial content was further shown not to decrease by measuring cytochrome oxidase activity (Table 1). A slight decrease of citrate synthase activity was assessed after 6-h incubation in the presence of glucose (Table 1). Altogether, these results show that repression of mitochondrial metabolism is not a prerequisite to promote cell proliferation.

**Table 2:** Hexokinase activity in wild type and mutant cells.

	**T0**	**T2**		**T4**		**T6**		**T8**	
	**–**	**–**	**+**	**–**	**+**	**–**	**+**	**–**	**+**
*S. c* – WT	UD	0.01 (± 0.02)	0.03 (± 0.03)	0.02 (± 0.03)	0.07 (± 0.05)	0.01 (± 0.02)	0.16 (± 0.04)*	0.01 (± 0.01)	0.21 (± 0.01)**
*S. c* – Δ*hxk2*	UD	0.01 (± 0.01)	0.09 (± 0.09)	0.01 (± 0.03)	0.36 (± 0.14)**	0.01 (± 0.02)	0.4 (± 0.16)**	UD	0.53 (± 0.30)**
*S. c* – Δ*hap4↑*	0.17 (± 0.07)	0.18 (± 0.09)*	0.18 (± 0.08)	0.29 (± 0.14)	0.44 (± 0.27)	0.3 (± 0.14)	0.45 (± 0.23)	0.32 (± 0.14)	0.38 (± 0.22)
*S. c* – Δ*hxk2hap4↑*	0.01 (± 0.01)	UD	0.08 (± 0.06)	UD	0.09 (± 0.04)	UD	0.13 (± 0.08)	UD	0.12 (± 0.05)

*Enzymatic activity was measured as stipulated in the Materials and Methods section and is expressed in μmol/min/mg protein. (**–**) indicates in absence of glucose. (+) indicates in the presence of glucose. T0-2-4-6-8 are the hours of incubation w/ or w/o glucose. Results shown are means of at least three separate experiments ± SD*.

*UD, Undetectable*.

### A Crabtree Negative Strain Exhibits an Increase in Growth Rate Without Any Mitochondrial Repression

**Figure 6:**
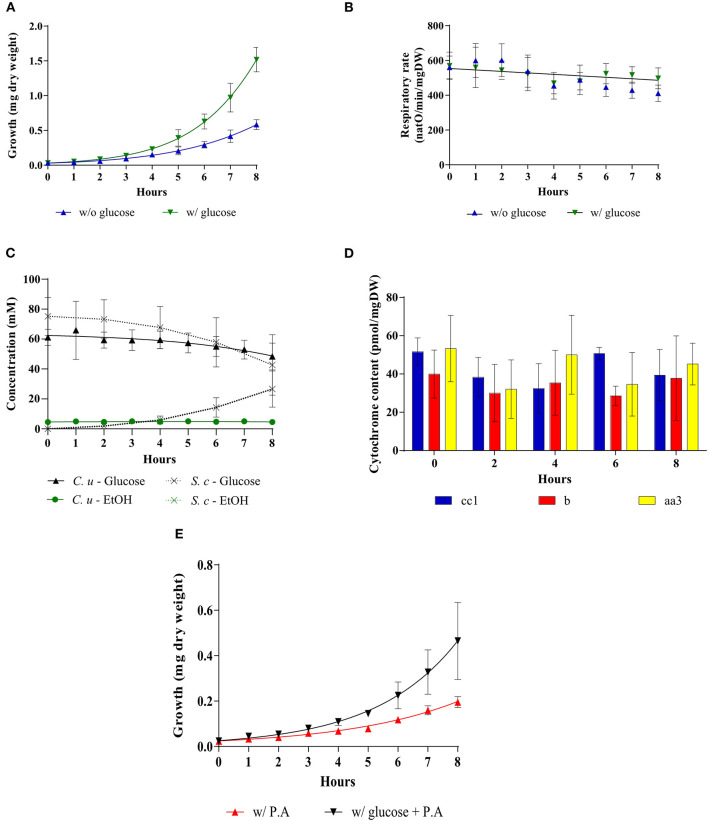
Addition of glucose to a Crabtree-negative strain, *Candida utilis*. The growth medium was supplemented with 60 mM of glucose at T0 (▼) or not (▲). **(A)** Growth was followed for 8 hours. Results shown represent means of at least 15 separate experiments ± SD. **(B)** The respiratory rate was followed for 8 h. Results shown represent means of at least 15 separate experiments ± SD. **(C)** Glucose consumption (• *C. utilis*) or (x *S. cerevisiae*) and ethanol production (• *C. utilis*) or (x *S. cerevisiae*) were quantified. Results shown are means of at least four separate experiments ± SD. **(D)** Cytochrome content was quantified every 2 h after glucose addition to the medium. Results shown represent means of at least five separate experiments ± SD. **(E)** For each condition, growth was followed for 8 h in the presence or absence of Piericidin A (200 μM). Results shown represent means of at least three separate experiments ± SD.

The unexpected above-mentioned results were obtained in a *Saccharomyces cerevisiae* mutant strain. We then sought to confirm these results (increase in growth rate in the presence of glucose with little/no decrease in mitochondrial respiration) with an alternate model. In the yeast community, yeast strains have long been characterized depending on their mitochondrial oxidative phosphorylation response to the presence of glucose. Crabtree-negative strains, also known as respiratory-obligatory strains are well-known as requiring an active mitochondrial metabolism for growth in the presence of glucose. Consequently, we made use of a yeast strain known not to exhibit glucose-induced repression of oxidative phosphorylation metabolism. In this *Candida utilis* strain, as previously shown with a *Saccharomyces cerevisiae* strain, glucose induces an increase in cell growth (Figure 6A). Mitochondrial respiratory rate is not significantly modified upon glucose addition although a slight decrease is observed in absence of glucose (Figure 6B). The increase in growth rate is associated with glucose consumption, albeit to a lower level than in the *Saccharomyces cerevisiae* strain (Figure 6C). EtOH production was minimal in this strain upon glucose addition in agreement with the maintenance of oxidative phosphorylation (Figure 6C). Further, no significant changes in mitochondrial cytochromes and mitochondrial enzymatic activities were observed upon glucose addition in this strain (Figure 6D and Table 1). Since *Candida utilis* exhibits a proton pumping complex I -which *Saccharomyces cerevisiae* does not exhibit-, similar experiments were done in the presence of Piericidin A, a complex I inhibitor (40–42), to ensure that glucose addition did not change the proton pumping stoichiometry of the respiratory chain which could lead to an increase in oxidative phosphorylation efficiency. Indeed, glycolysis will generate NADH that is reoxidized at the level of complex I. Since ATP/O is higher from complex I (2.5) than for all other dehydrogenases (1.5), one could imagine that glucose addition to these cells will increase oxidative phosphorylation efficiency (43–46). Figure 6E shows that the increase in growth rate assessed upon glucose addition occurs in the absence of a functional complex I, eluding an eventual role of oxidative phosphorylation efficiency change in the growth rate increase.

### There Is No Direct Link Between Oxidative Phosphorylation Repression and Cellular Growth Rate

Here, we developed a number of *S. cerevisiae* cellular models that allowed us to study the induction of the Warburg effect. As described above, these models exhibit various respiratory rate repression levels and various growth rates in the presence or absence of glucose. We calculated growth rates for every strain for the 4- to 8-h incubation time points since most changes in terms of growth and respiratory metabolism occur in the first 4 h. These growth rates were then plotted against the average respiratory rates for the same incubation time. This showed that there is not direct/simple link between oxidative phosphorylation repression and an increase in cell growth rate (data not shown but see results from Figures 1, 3, SD1 & SD2). Further, this occurs at a comparable coupling level between oxidation and phosphorylation at the level of the oxidative phosphorylation as shown by the constant ratio between spontaneous respiratory rate and non-phosphorylating respiratory rate (Table 3).

**Table 3:** Spontaneous and non-phosphorylating respiratory rates in wild type and mutant cells.

**Strains**	**JO_**2**_**	**JO_**2-non phosphorylating**_**	**Ratio**
*S. c* – WT	70 (± 4.5)	27 (± 0.8)****	2.59 (± 0.2)
*S. c* – Δ*hxk2*	91 (± 2.4)	36 (± 1.9)****	2.57 (± 0.4)
*S. c* – Δ*hap4↑*	122 (± 5.3)	50 (± 0.4)****	2.52 (± 0.2)
*S. c* – Δ*hxk2hap4↑*	192 (± 6.5)	73 (± 4.2)****	2.61 (± 0.2)
*C. u*	498 (± 14.6)	ND	ND

*Respiratory rates were assessed after 8-h incubation in the presence of glucose. Non-phosphorylating respiratory rate was determined in the presence of 0.1 mM Triethyltin, an inhibitor of mitochondrial ATPsynthase*.

*ND, not determined*.

### ATP Synthesis Flux Does Not Control Cellular Growth Rate

**Figure 7:**
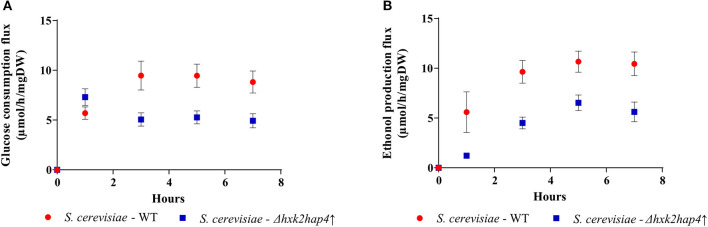
Glucose consumption and ethanol production fluxes. **(A)** The glucose consumption flux was calculated from glucose concentration in the culture medium and the amount of cells for each time point. Results shown represent means of at least four separate experiments ± SD. **(B)** The ethanol production flux was calculated from ethanol concentration in the culture medium and the amount of cells for each time point. Results shown represent means of at least four separate experiments ± SD.

The behavior of the Δ*hxk2hap4*↑ strain that is not typical of a Warburg effect raises a number of questions. Indeed, one of the proposed functions of the Warburg effect is an increased ATP synthesis flux through glycolysis allowing for an increased growth. We thus calculated glycolytic flux in both the wild type and Δ*hxk2hap4*↑ strain (data from Figures 1C, 5C). In the yeast *Saccharomyces cerevisiae*, the end product of “aerobic glycolysis” as defined by Warburg -that is fermentation- is EtOH (lactate in mammalian cells). Glucose consumption flux and EtOH production rate in the wild type (WT) and Δ*hxk2hap4*↑ strains are presented in Figures 7A,B, respectively. Glucose consumption flux reached a stationary state after about 4 h for both strains and this flux in the Δ*hxk2hap4*↑ strain is lower than that of the wild type strain (Figure 7A). EtOH production flux reached a stationary state after about 4 h for both strains and this flux in the Δ*hxk2hap4*↑ strain is lower than in the wild type strain (Figure 7B).

**Figure 8:**
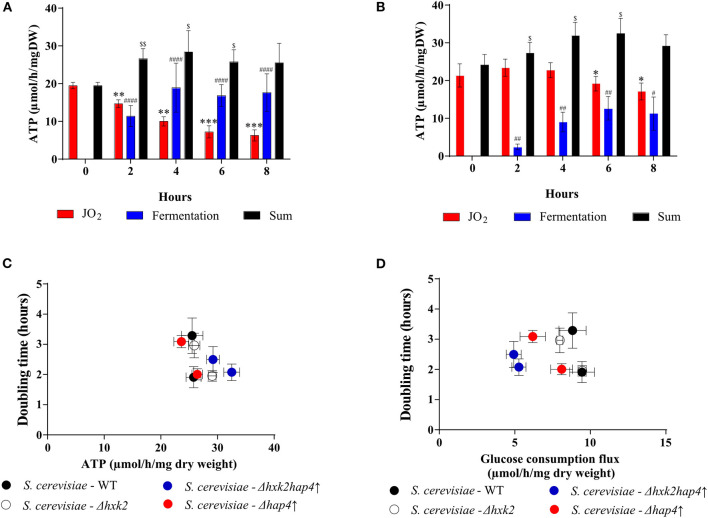
Effect of glucose on ATP synthesis flux in the different strains. ATP synthesis flux was calculated from the respiratory rate and the ethanol production flux according to the oxidation balance-sheet: ½ O_2_ = 1,5 ATP for respiratory rate; 1 EtOH = 2 ATP for fermentation to **(A)** *S. cerevisiae*- WT and **(B)** *S. cerevisiae -* Δ*hxk2hap4*↑. **(C)** Growth as a function of ATP synthesis flux at T6 and T8. **(D)** Growth as a function of glucose consumption flux at T6 and T8. For each strain, in the presence of glucose, doubling times were calculated between 4 and 6 h and then between 6 and 8 h after glucose addition in the medium. (•) *S. cerevisiae* WT, (•) Δ*hap4*↑, (°) Δ*hxk2***, **(•) Δ*hxk2hap4*↑. For each condition values are compared to the corresponding T0 and the *p*-value is represented as * for JO2; # for Fermentation; $ for Sum.

Upon fermentation, one mole of glucose allows for the production of two moles of EtOH. Since both glucose consumption flux and EtOH production flux are comparable in each strain after 4 h, only half the glucose is being fermented. Consequently, since 2 ATP are produced per fully metabolized glucose, under our conditions the glycolysis-linked ATP synthesis flux could be assimilated to EtOH production flux. The yeast Saccharomyces cerevisiae mitochondria lack a proton pumping complex I (47, 48). Thus, electron transfer through that respiratory chain always involves two proton pumping sites, the efficiency of the oxidative phosphorylation is assumed to be constant and about 1.5 ATP produced per oxygen (1/2 O_2_) consumed. Since we measured the respiratory rates of our strains throughout growth, we can estimate the ATP synthesis flux linked to mitochondrial oxidative phosphorylation. Mitochondria and glycolysis-derived ATP synthesis fluxes for the wild type and the mutant strains are presented in Figures 8A,B, respectively. In the wild type strain, upon glucose addition, a consequent decrease in mitochondria-linked ATP synthesis was associated with a concomitant increase in glycolysis-linked ATP synthesis (Figure 8A). In the Δ*hxk2hap4*↑ strain, glucose addition only slightly decreases mitochondria-derived ATP synthesis and glycolysis-linked ATP synthesis only accounts for about 30% of the total flux (Figure 8B). More importantly, there is no clear link between growth rate and ATP synthesis, whichever the origin of the ATP (Figure 8C). Further, if one plots cell growth vs. the glucose consumption flux, again, there is no clear link between these two parameters (Figure 8D).

## Conclusion

Throughout its history, the possible added value of the Warburg Effect has remained controversial and the potential function(s) of this energy metabolism rewiring remain(s) unclear. In this paper, we investigated the role of this rewiring in promoting cell growth and division. We made use of yeast to study the induction of the Warburg effect and its kinetics. Further, the use of mutants allowed us to further study the relationship between growth rate and the two pathways that support ATP synthesis namely glycolysis and oxidative phosphorylation. We show that yeast is a good model to study the Warburg effect, since all three parameters and their modulation by glucose can be reconstituted upon glucose addition to cells. Otto Warburg proposed that the decrease in oxidative phosphorylation activity was due to dysfunctional mitochondria. Here, we show that in our model upon induction of the Warburg effect, the decrease of mitochondrial oxidative phosphorylation is not due to dysfunctional mitochondria but to a decrease in mitochondrial biogenesis. Upon growth and while mitochondrial biogenesis is strongly decreased, mitochondria are diluted in the daughter cells. This result is in good agreement with a number of studies showing that mitochondrial are not dysfunctional in a number of cancer cells since their oxidative phosphorylation activity can be increased (4). However, this does not preclude that in some cases mitochondria cannot be dysfunctional since it has been shown that in some cancer cell line, succino-dehydrogenase was mutated and mostly inactive (49–52). The point here is that there is no need for mitochondria to be dysfunctional to evidence a Warburg effect. We next investigated the link between oxidative phosphorylation repression and the increase in growth rate. We were able to uncouple growth rate and oxidative phosphorylation repression by deleting Hxk2p and overexpressing Hap4p, indicating that both the maintenance of mitochondrial biogenesis and the loss of *Hxk2* function are necessary for this uncoupling. Since glycolysis is maintained in Δ*hxk2* cells, it is tempting to speculate that the transcription factor function of *hxk2* is involved here. The use of mutant cells as well as Crabtree negative cells allowed us to show that there is no direct link between the decrease in oxidative phosphorylation activity and the increase in growth rate. Indeed, when oxidative phosphorylation activity is maintained upon glucose addition to cells there is a clear increase in growth rate, indicating no correlation between both parameters. This indicates that growth rate is not controlled by the modulation of energetic metabolism but rather depends on the presence of glucose. Whether glucose here acts as a signaling molecule or as a substrate of biosynthetic pathways remains to be determined. However, this is no easy task since it seems that in terms of fluxes, the flux through biosynthetic pathways is minor compared to the flux through glycolysis. Last, ATP synthesis fluxes in the presence of absence of a Warburg effect were calculated in our cellular models from fermentation and oxidative phosphorylation fluxes. We were able to show that ATP synthesis fluxes do not control cell growth. This experimental result is in accordance with previous calculations that suggest that the amount of ATP required for cell growth and division may be far lower than that required for basal cellular maintenance (53). Consequently, one can conclude that the growth-promoting role of the Warburg effect does not go through an increase in ATP synthesis fluxes.

In recent studies tumor energy metabolism has been assessed under more physiological concentrations of glucose and hypoxic conditions. It has been shown that lower mitochondrial biogenesis, deficient HIF-1α/mutant p53 interaction, and development of a pseudohypoxic state under normoxia were the apparent biochemical mechanisms underlying glycolysis activation and OxPhos downregulation in HeLa-M cells (54). Further, decreasing glucose concentration down to 2.5 mM restrains the Warburg phenotype, in hypoxic HeLa cell cultures and microspheroids (55). This clearly indicates that both parameters are crucial for the study of the Warburg effect in mammalian cells and should be considered more often.

In this paper, we show that the cell energy metabolism reorganization observed upon de Warburg effect is not mandatory for an increase in cell growth. Further, maintenance of oxidative phosphorylation activity does not affect the glucose growth promoting effect that occurs at various glycolysis rate. Last, cell ATP synthesis flux is shown not to control the growth rate. A number of reports in human cancer cells have pointed out a quite variable mitochondrial content in these cells. Indeed, while some studies demonstrate a reduction of oxidative phosphorylation capacity in different types of cancer cells, other investigations revealed contradictory modifications with the upregulation of oxidative phosphorylation components and a larger dependency of cancer cells on oxidative energy substrates for anabolism and energy production (56). Studying these cells in terms of cell growth and ATP synthesis flux in normoxia and normoglycemia would be of great interest to unravel the added value of the Warburg effect in cell proliferation.

## Data Availability Statement

All datasets generated for this study are included in the article/Supplementary Material.

## Author Contributions

CB, NH, and SC designed and performed the experiments. CB, NH, SC, MR, SR, and AD analyzed the corresponding results. CB and NH wrote the paper with SR, MR, and AD. All authors contributed to the article and approved the submitted version.

## Conflict of Interest

The authors declare that the research was conducted in the absence of any commercial or financial relationships that could be construed as a potential conflict of interest.

## Supplementary Material

Supplementary Figure 1 Induction of the Warburg effect in *S. cerevisiae* Δ*hxk2*. The growth medium of *S. cerevisiae* was supplemented with 60 mM of glucose at T0 (■ Δ*hxk2*) and (**x** WT) or not (• Δ*hxk2*) and (+ WT). **(A)** Growth was followed for 8 h. Results shown are means of at least five separate experiments ± SD. **(B)** The respiratory rate was followed for 8 h. Results shown represent means of at least five separate experiments ± SD. **(C)** Glucose consumption (• Δ*hxk2*) or (**x** WT) and ethanol production (▲ Δ*hxk2*) or (**x** WT) were quantified. Results shown represent means of at least seven separate experiments ± SD.

Supplementary Figure 2 Induction of the Warburg effect in *S. cerevisiae* Δ*hap4*↑. The growth medium of *S. cerevisiae* was supplemented with 60 mM of glucose at T0 (■ Δ*hap4*↑) and (**x** WT) or not (• Δ*hap4*↑) and (+ WT). **(A)** Growth was followed for 8 h. Results shown are means of at least five separate experiments ± SD. **(B)** The respiratory rate was followed for 8 h. Results shown represent means of at least five separate experiments ± SD. **(C)** Glucose consumption (• Δ*hap4*↑) or (**x** WT) and ethanol production (▲ Δ*hap4*↑) or (**x** WT) were quantified. Results shown represent means of at least seven separate experiments ± SD.

## Abbreviations

ATP: Adenosine triphosphate
ΔGp: phosphate potential
ΔG_redox_: redox potential
O_2_: dioxygen
HAP: heme activator protein
Hxk: Hexokinase
CCCP: carbonyl cyanide m-chlorophenylhydrazone
KOH: Potassium hydroxide
NAD^+^: Nicotinamide adenine dinucleotide
PCA: Perchloric acid
NaOH: Sodium hydroxide
SDS: Sodium dodecyl sulfate
OD: Optical density
PGK1: phosphoglycerate kinase 1
HCl: hydrochloric acid
PiK: Potassium phosphate
EtOH: Ethanol
WT: Wild Type
w/: with
w/o: without.
