# Supplementary figures and images for: The Warburg Effect in Yeast: Repression of Mitochondrial Metabolism Is Not a Prerequisite to Promote Cell Proliferation
Source: Front Oncol. 2020 Aug 19;10:1333. doi: 10.3389/fonc.2020.01333 (PMC7466722; doi:10.3389/fonc.2020.01333)

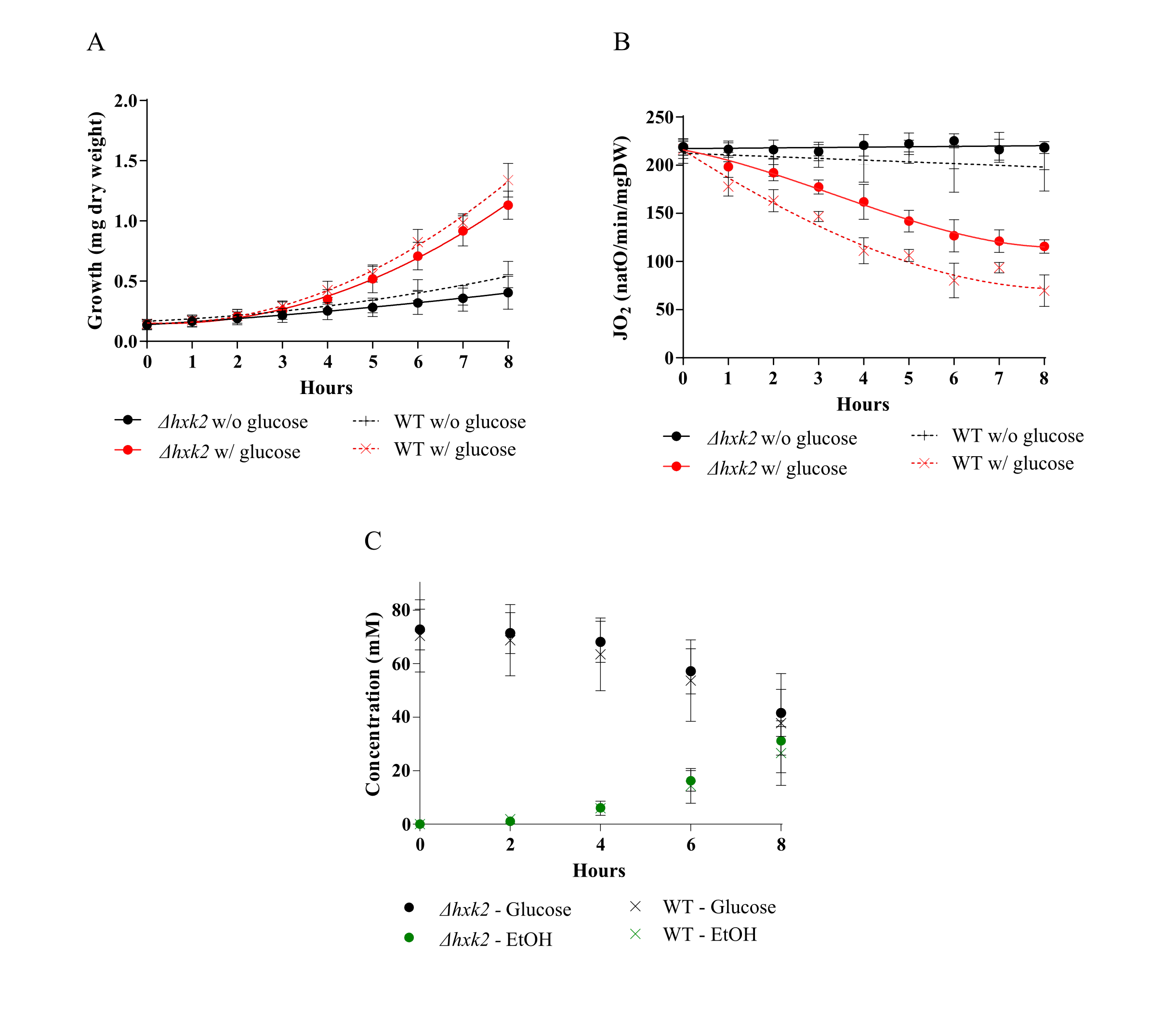

Supplement: Supplementary Figure 1 — Induction of the Warburg effect in S. cerevisiae Δhxk2. The growth medium of S. cerevisiae was supplemented with 60 mM of glucose at T0 (■ Δhxk2) and (x WT) or not (• Δhxk2) and (+ WT). (A) Growth was followed for 8 h. Results shown are means of at least five separate experiments ± SD. (B) The respiratory rate was followed for 8 h. Results shown represent means of at least five separate experiments ± SD. (C) Glucose consumption (• Δhxk2) or (x WT) and ethanol production (▲ Δhxk2) or (x WT) were quantified. Results shown represent means of at least seven separate experiments ± SD. [file Image_1.tiff]

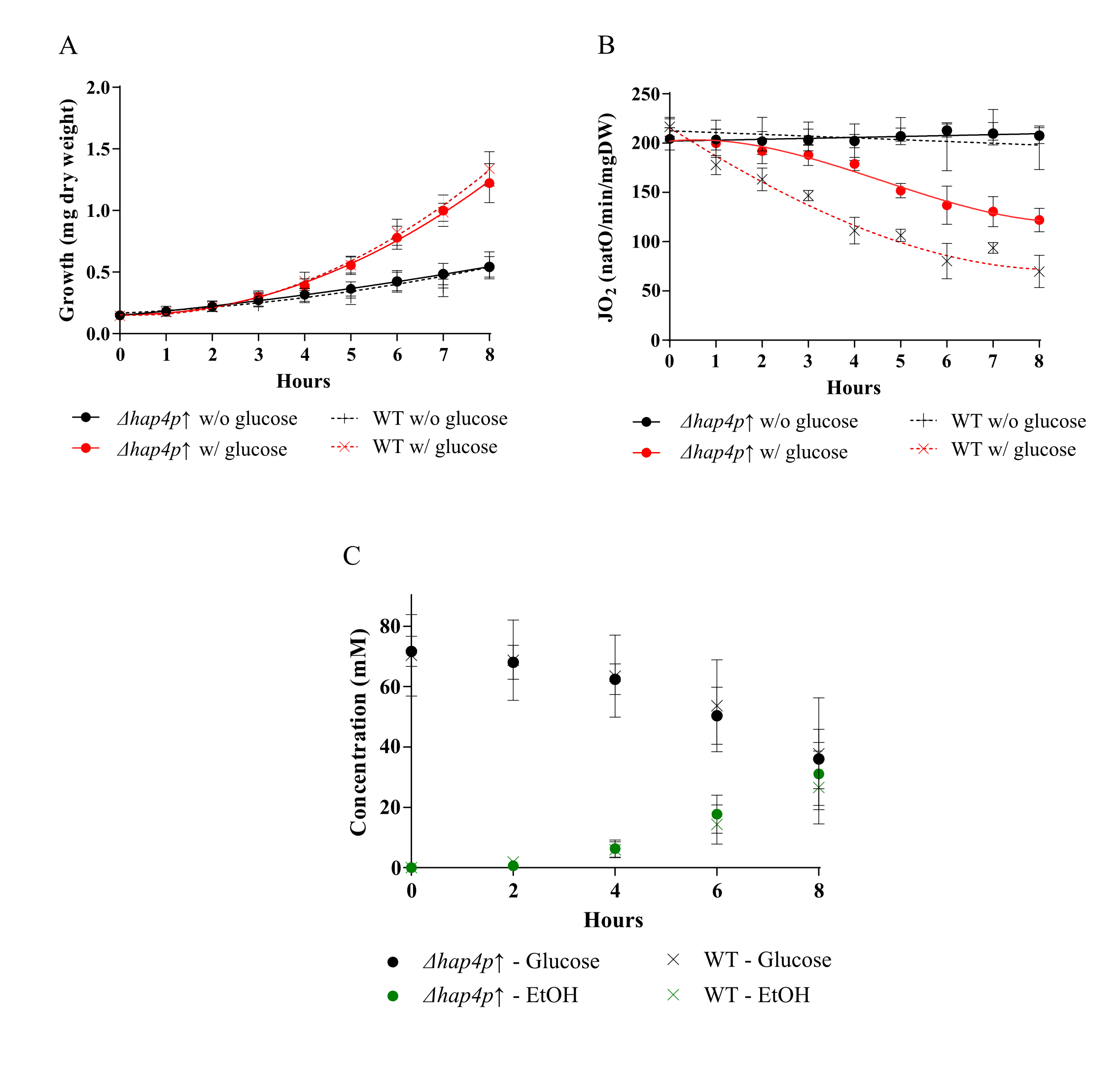

Supplement: Supplementary Figure 2 — Induction of the Warburg effect in S. cerevisiae Δhap4↑. The growth medium of S. cerevisiae was supplemented with 60 mM of glucose at T0 (■ Δhap4↑) and (x WT) or not (• Δhap4↑) and (+ WT). (A) Growth was followed for 8 h. Results shown are means of at least five separate experiments ± SD. (B) The respiratory rate was followed for 8 h. Results shown represent means of at least five separate experiments ± SD. (C) Glucose consumption (• Δhap4↑) or (x WT) and ethanol production (▲ Δhap4↑) or (x WT) were quantified. Results shown represent means of at least seven separate experiments ± SD. [file Image_2.tiff]
